# Multifunctional Roles of Magnesium Ions in Modulating Bone Regeneration-Related Cells

**DOI:** 10.3390/bioengineering13091093

**Published:** 2026-09-21

**Authors:** Xiangcheng Lei, Ruipeng Li, Yangyi Nie, Deju Gao, Wei Zhang, Yuxiao Lai, Cairong Li

**Affiliations:** 1 Centre for Translational Medicine Research and Development, Shenzhen Institutes of Advanced Technology, Chinese Academy of Sciences, Shenzhen 518055, Chinayx.lai@siat.ac.cn (Y.L.); 2 Faculty of Health Sciences, University of Macau, Macau 999078, China; 3 University of Chinese Academy of Sciences, Beijing 101408, China

**Keywords:** magnesium ions, bone regeneration, osteogenesis, angiogenesis, neuroregulation, immunomodulation

## Abstract

Magnesium ions (Mg^2+^) play pivotal roles in bone regeneration by regulating the biological functions of multiple bone repair-related cells. However, the effective concentration ranges and optimal concentration of Mg^2+^ for different cell types remain incompletely defined, which limits the precise design of Mg-containing biomaterials with controllable ion release for repairing of bone defects. In this study, the concentration-dependent effects of Mg^2+^ on the biological functions of bone regeneration-related cells were systematically investigated. Human bone marrow mesenchymal stem cells (hBMSCs), human umbilical vein endothelial cells (HUVECs), rat Schwann cells (RSCs), and RAW264.7 macrophage were selected to evaluate the effective concentration of Mg^2+^ for promoting osteogenesis, angiogenesis, neuroregulation, and immunomodulation, respectively. The results demonstrated that Mg^2+^ concentrations ranging from to 16 mM, particularly 8 mM, significantly upregulated the expression of M2 macrophage-related markers, thereby promoting the polarization of RAW264.7 macrophages toward the anti-inflammatory M2 phenotype. Moreover, Mg^2+^ concentrations between 2 and 8 mM effectively enhanced the osteogenic activity of hBMSCs, the angiogenic activity of HUVECs, and the neuroregulatory function of RSCs, as confirmed by immunofluorescence staining and quantitative PCR analysis of osteogenic, angiogenic, and neuroregulation-related marker expression. These findings provide a comprehensive understanding of the concentration-dependent biological effects of Mg^2+^ on bone regeneration-related cells and establish a theoretical basis for the rational design of next-generation Mg^2+^-releasing biomaterials with precise biological regulation.

## 1. Introduction

Bone defect repair remains a major challenge in clinical orthopedics and regenerative medicine, particularly under complex pathological conditions such as large segmental defects, trauma, and osteoporosis, where complete bone regeneration is difficult to achieve [1,2,3,4,5]. Although bone tissue possesses intrinsic regenerative capacity, the repair process is often slow and accompanied by incomplete restoration of structure and function. In recent years, the development of bone tissue engineering has provided new strategies for bone regeneration. However, accumulating evidence suggests that promoting osteogenesis alone is insufficient to meet the demands of complex bone repair, and that angiogenesis, neural regulation, and immune microenvironment remodeling play equally critical roles in this process [6,7].

Recent studies indicate that the performance of bone regeneration-related biomaterials depends on material composition, microstructure, degradation behavior, and controlled release of bioactive components. Rolling reduction of Zn–Mg alloys and hydroxyapatite/zinc oxide nanoparticle-reinforced glass ionomer cement highlight the importance of structural optimization and inorganic bioactive components in improving mechanical, and osteogenic properties [4,5]. In addition, acid-neutralizing metal–phenolic microspheres demonstrate the value of microenvironment-responsive release systems for regulating inflammation and tissue degeneration [8]. In this context, our study provides preliminary in vitro evidence that different bone regeneration-related cells require different Mg^2+^ concentration ranges, suggesting that future Mg^2+^ containing biomaterials should emphasize controlled and stage-specific Mg^2+^ release to better support bone repair.

Magnesium (Mg), an essential divalent cation in the human body, is predominantly stored in bone tissue (~60%) and plays a crucial role in bone metabolism and various enzymatic reactions. As an important component of bone tissue, magnesium ions (Mg^2+^) not only participate in bone matrix mineralization but also regulate cellular behaviors involved in bone formation and remodeling [9,10,11,12]. With the rapid development of biodegradable magnesium-based biomaterials, Mg^2+^ has attracted increasing attention due to its excellent biocompatibility and bioactivity [13,14]. In this study, MgCl_2_ was used as the Mg^2+^ supplement because its high solubility enables the accurate and reproducible preparation of media containing defined Mg^2+^ concentrations while causing minimal changes in pH. This approach also avoids the uncertainties and potential confounding effects associated with the degradation of magnesium-containing biomaterials, including changes in pH and osmolality and the release of other degradation products. Numerous studies have demonstrated that Mg^2+^ can significantly promote the proliferation, migration, and osteogenic differentiation of bone marrow mesenchymal stem cells (BMSCs), accompanied by the upregulation of osteogenesis-related markers [15,16]. Mechanistically, Mg^2+^ is involved in bone formation through the regulation of multiple signaling pathways, including Notch, Wnt/β-catenin, PI3K/Akt/mTOR, and MAPK pathways [17,18,19,20,21,22].

In addition to its direct osteogenic effects, the role of Mg^2+^ in angiogenesis has also been increasingly recognized. During bone regeneration, the formation of vascular networks is essential for nutrient supply and cell recruitment. Previous studies have shown that Mg^2+^ can promote endothelial cell migration and vascular formation by upregulating key angiogenic factors such as vascular endothelial growth factor (VEGF) and hypoxia-inducible factor-1α (HIF-1α) [17,23,24,25,26]. Furthermore, Mg^2+^ can enhance the secretion of platelet-derived growth factor-BB (PDGF-BB) from osteoblasts, thereby coupling osteogenesis with angiogenesis and significantly improving bone regeneration outcomes [27,28,29,30,31]. These findings identify Mg^2+^ as a critical regulator that coordinates osteogenesis and angiogenesis. Previous studies have shown that endothelial cells, especially primary HUVECs, are sensitive to Mg^2+^ exposure. For example, recent regression-based analysis demonstrated that Mg^2+^ concentrations above 2 mM could exert detrimental effects on sensitive primary HUVECs [32,33]. However, some studies also found that 10 mM Mg^2+^ exhibits a significant pro-angiogenic effect in endothelial cell migration and tubule formation assays [34]. These findings indicate that the endothelial response to Mg^2+^ is highly context dependent.

The immune microenvironment, particularly macrophage polarization, plays a pivotal role in bone repair. Mg^2+^ can modulate immune responses by inhibiting inflammatory signaling pathways such as NF-κB, suppressing the release of pro-inflammatory cytokines, and promoting macrophage polarization toward the anti-inflammatory M2 phenotype [35]. These effects collectively contribute to the establishment of a pro-regenerative microenvironment conducive to bone healing [23,36,37,38,39]. In addition, Mg^2+^ can regulate the crosstalk between macrophages and BMSCs, further enhancing osteogenic differentiation. This emerging “immunoregulation–bone regeneration” axis further underscores the therapeutic potential of Mg^2+^ in bone tissue engineering [40,41,42,43,44].

The role of the nervous system in bone regeneration has attracted increasing attention in recent years. Previous studies have shown that magnesium plays important roles in nerve transmission, neuromuscular conduction, and protection against excitotoxicity-related neuronal injury, suggesting its potential relevance in neurological regulation and repair [45]. Moreover, Mg-based materials have recently been investigated for neurological applications. For example, Mg-Li thin films showed favorable glial cytocompatibility and did not markedly induce neuroinflammatory activation in an in vitro brain slice model, indicating that Mg-containing materials may provide a permissive microenvironment for neural-related applications [46]. Neural innervation not only influences bone metabolism but also participates in bone remodeling through the release of neuropeptides. Recent studies have shown that Mg^2+^ can promote neural function recovery by regulating neuro-related signaling pathways and indirectly enhance bone regeneration via the “neuro–bone coupling” mechanism [47,48,49,50]. Moreover, Mg^2+^ release has been reported to facilitate neurite outgrowth and the expression of neurotrophic factors, thereby exerting synergistic effects in bone defect repair [51,52,53,54,55,56,57].

Despite the multifunctional roles of Mg^2+^ in osteogenesis, angiogenesis, immunomodulation, and neuroregulation, its biological effects are highly concentration dependent. Previous studies have demonstrated that appropriate concentrations of Mg^2+^ can promote cell proliferation and differentiation, whereas excessive concentrations may inhibit cellular activity and even induce cytotoxicity. Different cell types involved in bone regeneration may respond to distinct Mg^2+^ concentration ranges. Therefore, defining the effective concentration ranges and optimal concentration of Mg^2+^ in a multicellular system and elucidating its multidimensional regulatory function are significant for its precise application in bone tissue engineering [58,59,60].

**Figure 1:**
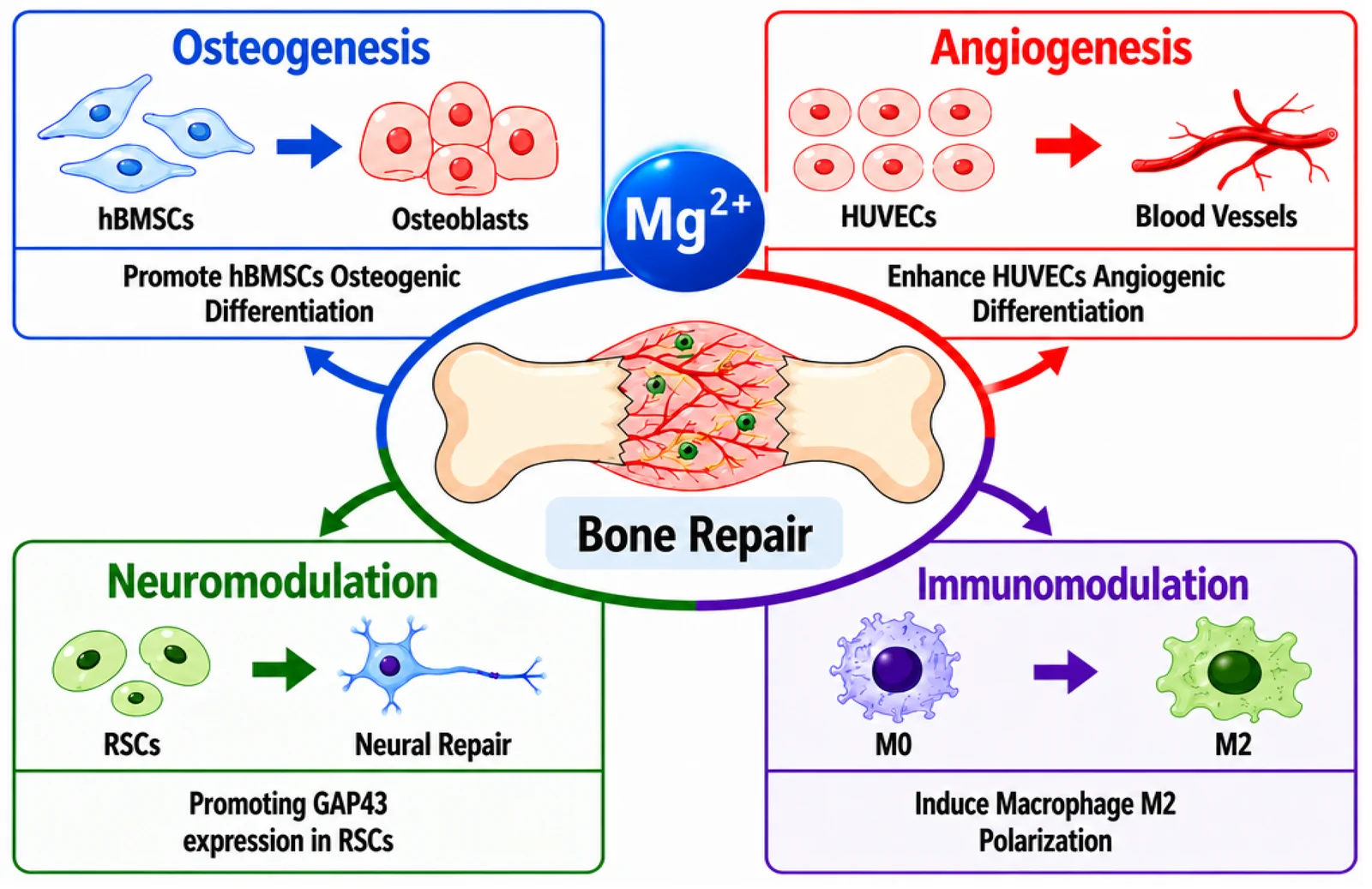
Schematic illustration of multifunctional roles of magnesium ions in modulating bone regeneration-related cells.

In summary, the present study systematically investigated the multifunctional regulatory roles of Mg^2+^ in bone regeneration by establishment of a concentration-gradient model. In this study, culture media containing different concentrations of Mg^2+^ were prepared by supplementing magnesium chloride (MgCl_2_). Then the effects of Mg^2+^ on hBMSCs, HUVECs, RSCs, and RAW264.7 were comprehensively evaluated (Figure 1). Through the analysis of cell proliferation, osteogenic differentiation, angiogenesis, macrophage polarization, and neuro-related functions, this study provides experimental reference information for controlling Mg^2+^ release in biomaterial design and for achieving stage-specific regulation of bone repair-related cellular events.

## 2. Materials and Methods

### 2.1. Cell Culture

Representative cell models were used for assessing a distinct aspect of biomaterial performance. hBMSCs were cultured in α-MEM (Gibco, Grand Island, NY, USA) supplemented with 10% fetal bovine serum (FBS, Gibco, USA) and 1% penicillin–streptomycin (Gibco, USA) at 37 °C in a humidified atmosphere containing 5% CO_2_. Cells at passages 3–5 were used for subsequent experiments. RAW264.7, RSCs, and HUVECs were cultured in DMEM (Gibco, USA) supplemented with 10% FBS and 1% penicillin–streptomycin (Gibco, USA) under the same culture condition. MgCl_2_ was used to prepare culture media with different Mg^2+^ concentrations (0, 2, 4, 8, 16, and 32 mM). Solutions were sterilized using a 0.22 μm filter and stored at 4 °C before use. All the cells were purchased from Procell Company, Wuhan, China (Table 1).

### 2.2. CCK-8 Assay

Cells were seeded in 96-well plates at a density of 5 × 10^3^ cells/well. After attachment, the medium was replaced with culture medium containing different concentrations of Mg^2+^. At 1, 3, and 7 days of culture, Cell Counting Kit-8 (CCK-8, Mikx, Shenzhen, China) reagent was added and incubated at 37 °C for 1 h. The absorbance at 450 nm was measured using a microplate reader (Allsheng, Hangzhou, China).

### 2.3. Live/Dead Staining

Cells were seeded in 24-well plates at a density of 1 × 10^5^ cells/well and treated with Mg^2+^ at indicated concentrations. At designated time points (1, 3, and 7 days), cells were stained with Calcein AM/PI (Beyotime, Shanghai, China) for 30 min in the dark. Fluorescence images were captured using a fluorescence microscope. Green fluorescence indicated live cells, whereas red fluorescence indicated dead cells.

### 2.4. Immunofluorescence Staining

RAW264.7 cells, seeded in confocal dishes at a density of 5 × 10^4^ cells/well, were stimulated with 200 ng/mL LPS for 24 h and then treated with different concentrations of Mg^2+^ for another 24 h. Cells were fixed with 4% paraformaldehyde, permeabilized with 0.1% Triton X-100, and blocked before incubation with primary antibodies against iNOS, TNF-α, IL-10 and CD206 (1:200, HUABIO, Hangzhou, China) at 4 °C overnight. After incubation with fluorescent secondary antibodies, nuclei were stained with DAPI. Images were acquired using a confocal laser scanning microscope (Zen, Oberkochen, Germany). Similarly, RSCs were treated with gradient concentrations of Mg^2+^ 24 h, followed by immunofluorescence staining for GAP43, S100β, and NF200. BMSCs were treated with gradient concentrations of Mg^2+^ 24 h, followed by immunofluorescence staining for RUNX2 and OCN. Details of the antibodies are provided in Table 2.

### 2.5. Tube Formation Assay of HUVECs

To investigate the effect of Mg^2+^ on the angiogenic capacity of HUVECs, a tube formation assay was performed. Matrigel was thawed at 4 °C overnight and added to pre-cooled 96-well plates. After polymerization at 37 °C for 30 min, HUVECs were seeded onto the Matrigel-coated wells at a density of 2 × 10^4^ cells/well and cultured in medium supplemented with different concentrations of Mg^2+^ ranging from 0 to 32 mM. After 4 h of incubation, capillary-like tube formation was observed using an inverted microscope. The tube formation assay was quantified using ImageJ (Version 1.51j8) software with the Angiogenesis Analyzer plugin. The tube-like structures were identified by the plugin, and quantitative parameters, including total tube length, number of nodes, number of junctions, number of meshes, and number of branches, were derived from the analyzed images.

### 2.6. Cell Migration Assay of HUVECs

HUVECs were seeded in 12-well plates at a density of 2 × 10^5^ cells/well and grown to confluence. A scratch was created using a sterile pipette tip, and cells were washed with PBS to remove debris. Fresh medium containing different concentrations of Mg^2+^ was added. Images were captured at 0, 12, and 24 h. Migration distance was quantified using ImageJ software.

### 2.7. Osteogenic Induction of hBMSCs

Osteogenic induction medium was prepared with 10% FBS, 1% penicillin-streptomycin, 10 mM β-glycerophosphate, 50 μg/mL ascorbic acid, and 100 nM dexamethasone (all from Sigma-Aldrich, St. Louis, MO, USA). hBMSCs were seeded in 12-well plates at a density of 2 × 10^5^ cells/well. When cell confluence reached 70–80%, the complete medium was replaced with osteogenic induction medium containing different concentrations of Mg^2+^. The medium was changed every 3 days, and cells were cultured for 7, 14, or 21 days for further analysis.

### 2.8. ALP Staining, ALP Activity, and ARS Staining

hBMSCs were cultured in osteogenic medium for 7 and 14 days and stained using an ALP staining kit (Beyotime, China) according to the manufacturer’s instructions. ALP activity was measured using an ALP assay kit (Beyotime, China). Cell lysates were collected, and absorbance was measured at 520 nm.

Alizarin Red S (ARS) staining was performed after 14 or 21 days of osteogenic induction of hBMSCs. Cells were fixed with 4% paraformaldehyde for 15 min, washed with deionized water, and stained with 2% ARS solution (pH 4.2, OriCell, Guangzhou, China) for 20 min at room temperature. Then mineralized nodules were observed and imaged under a microscope. The amount of calcium deposition was quantified by destaining with 10% cetylpyridinium chloride monohydrate (CPC) at room temperature for 30 min (*n* = 3), and the absorbance of the solution was measured at 562 nm.

### 2.9. Quantitative Real-Time PCR (qRT-PCR)

For qRT-PCR analysis, hBMSCs, HUVECs, and RSCs were seeded in 12-well plates at a density of 2 × 10^5^ cells/well. After 24 h of Mg^2+^ treatment, total RNA was extracted from hBMSCs, HUVECs, and RSCs. Total RNA was extracted using TRIzol reagent (Invitrogen, Carlsbad, CA, USA) and reverse-transcribed into cDNA. Quantitative PCR was performed using a SYBR Green kit on an Roche LC96 system (Roche, Rotkreuz, Switzerland). The expression levels of Runx2, Osx, Col1a1, Bmp2 of hBMSC were detected, with GAPDH as the internal control. Meanwhile, the expression levels of Vegf-a, Hif1α, Nos3 of HUVEC were detected, with GAPDH as the internal control. The expression levels of Tuj1, Map2, Nestin of RSC were detected, with rat-GAPDH as the internal control. Relative gene expression was calculated using the 2^−ΔΔCt^ method and the primers are listed in Table 3.

### 2.10. Flow Cytometry Analysis

RAW264.7 were seeded in 12-well plates at a density of 2 × 10^5^ cells/well and stimulated with 200 ng/mL LPS (Macklin, Shanghai, China) for 24 h to establish an inflammatory macrophage model in vitro. After LPS stimulation, the cells were treated with different concentrations of Mg^2+^ for an additional 24 h. Subsequently, the cells were collected, washed with PBS, and incubated with fluorochrome-conjugated antibodies against CD86 (CL488-98025, Proteintech, Wuhan, China) and CD206 (CL594-98031, Proteintech) at 4 °C in the dark. After staining, the cells were washed and resuspended in PBS, and the expression levels of CD86 and CD206 were analyzed by flow cytometry.

### 2.11. Statistical Analysis

All experiment groups were performed with three technical replicates within a single experiment. Data are presented as mean ± standard deviation (SD). Statistical analysis was conducted using GraphPad Prism (Version 9.0.0) software. Differences between two groups were analyzed using Student’s *t*-test, while multiple group comparisons were performed using one-way ANOVA. The value of *p* < 0.05 was considered statistically significant.

## 3. Results

### 3.1. Biocompatibilities of Different Concentrations of Mg^2+^ for Bone Repair-Related Cells

**Figure 2:**
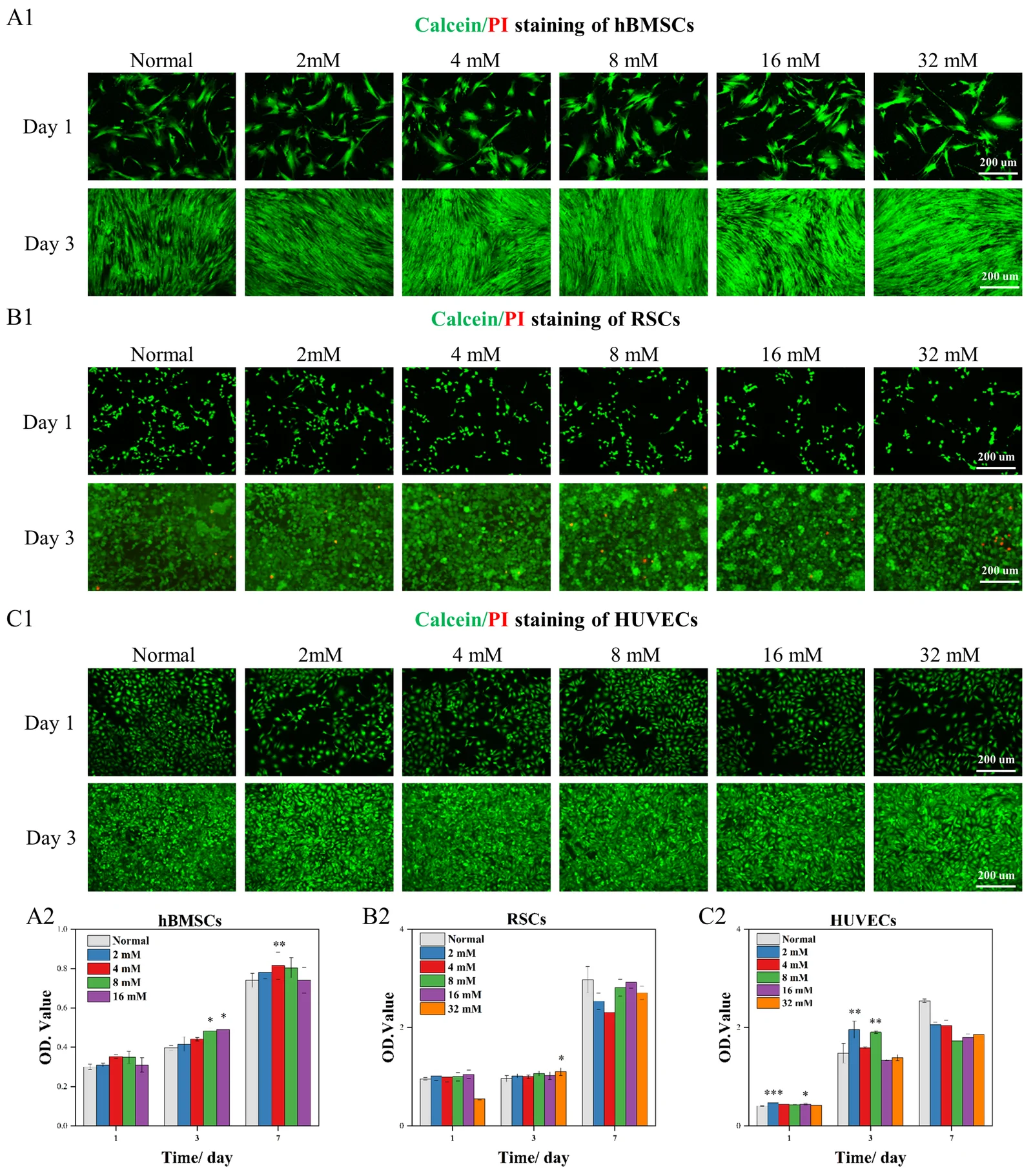
Cell adhesion and proliferation of different cells treated with various concentrations of Mg^2+^ (0–32 mM). Live/dead staining images and CCK-8 assay of hBMSCs (**A1**,**A2**), RSCs (**B1**,**B2**), and HUVECs (**C1**,**C2**). Green fluorescence indicates live cells, whereas red fluorescence indicates dead cells. Data are presented as mean ± SD. *n* = 3. *, significant difference compared to the Normal group, * *p* < 0.05, ** *p* < 0.01, *** *p* < 0.001.

The live (green)/dead (red) cell staining images of hBMSCs cultured with additional Mg^2+^ (0–32 mM) showed that there are no obvious dead cells observed when the concentrations of Mg^2+^ lower than 16 mM (Figure 2(A1)). CCK-8 assays demonstrated that Mg^2+^ significantly promoted the proliferation of hBMSCs at days 1 and 3 in both a time- and concentration-dependent manner. Compared with the control group, Mg^2+^ at concentrations lower than 8 mM can promote cell proliferation at all time points. In contrast, higher concentrations of Mg^2+^ (16 and 32 mM) showed a reduced promotive effect and even a slight inhibitory trend (Figure 2(A2)). Mg^2+^ exhibited a distinct time-dependent effect on RSCs adhesion and proliferation (Figure 2(B1,B2)). Live/dead staining of RSCs showed consistent results, where Mg^2+^ at 2–8 mM promoted cell viability at early time points. At day 1 and 3, Mg^2+^ (2–8 mM) significantly promoted RSCs proliferation. However, by day 7, this effect was reversed, and Mg^2+^ treatment resulted in an overall inhibitory trend. Notably, the inhibitory effect was less pronounced at lower concentrations (2 and 4 mM), whereas higher concentrations (≥8 mM) led to more obvious suppression of cell proliferation. Live/dead staining images of HUVECs showed good cytocompatibility within the tested concentration range and increased proliferative activity at the early stage, while prolonged exposure was associated with a reduced proliferative response. (Figure 2(C1)). CCK-8 assays further revealed a time-dependent regulatory effect of Mg^2+^ on HUVEC proliferation. At day 1, Mg^2+^ treatment increased the OD values compared with the control group, suggesting that Mg^2+^ could promote HUVEC proliferation at the early stage. However, at days 3 and 7, the OD values decreased after Mg^2+^ stimulation, indicating that prolonged Mg^2+^ treatment may reduce the proliferative activity of HUVECs. These results suggest that Mg^2+^ has few cytotoxic effects on HUVECs (Figure 2(C2)). Overall, Mg^2+^ exhibited a concentration- and time-dependent regulatory effect on hBMSCs, RSCs, and HUVECs. The concentration of Mg^2+^ lower than 16 mM showed little cytotoxicity and promoted cell proliferation at the early stage.

### 3.2. Regulatory Effects of Mg^2+^ on Macrophage Polarization

To investigate the immunomodulatory effects of Mg^2+^ on macrophage polarization, an inflammatory model was established by stimulating RAW264.7 with LPS (200 ng/mL). The cells were subsequently treated with different concentrations of Mg^2+^. After 24 h of treatment, immunofluorescence staining was performed to evaluate the expression of M1 (pro-inflammatory) and M2 (anti-inflammatory) macrophage markers.

**Figure 3:**
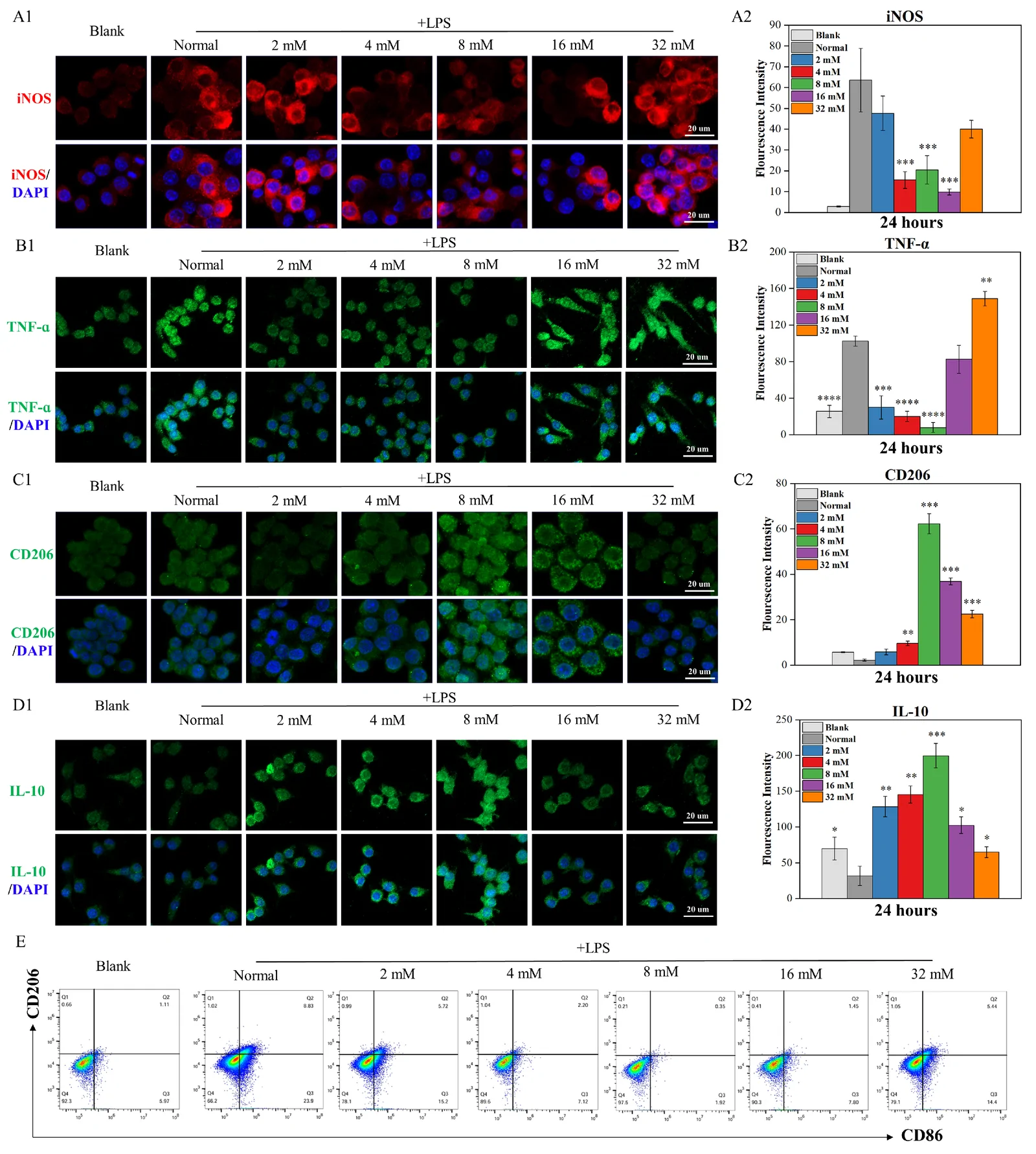
Regulatory effects of Mg^2+^ on macrophage polarization. Representative immunofluorescence images of iNOS (red, (**A1**)), TNF-α (green, (**B1**)), CD206 (green, (**C1**)), and IL-10 (green, (**D1**)) of RAW264.7 cells cultured with Mg^2+^ for 24 h, together with quantitative fluorescence intensity analysis, DAPI (blue) indicates cell nucleus (**A2**–**D2**). Flow cytometry was further used to evaluate CD86 and CD206 expression in RAW264.7 cells after different Mg^2+^ treatments for 24 h (**E**). Data are presented as mean ± SD. *n* = 3. *, significant difference compared to the Normal group, * *p* < 0.05, ** *p* < 0.01, *** *p* < 0.001, **** *p* < 0.0001.

Compared with the Normal group, the expression of the M1 macrophage marker iNOS (red fluorescence) showed a decreasing trend following treatment with 4–16 mM Mg^2+^ (Figure 3(A1,A2)). In addition, immunofluorescence staining of the pro-inflammatory cytokine TNF-α (green fluorescence) showed significantly downregulated after treatment with 2–16 mM Mg^2+^ compared with the Normal group (Figure 3(B1,B2)).

In contrast, the expression of the M2 macrophage marker CD206 (green fluorescence) exhibited an opposite pattern. CD206 expression was significantly upregulated under moderate Mg^2+^ concentrations (4–32 mM), with the most pronounced increase observed in 8 mM Mg^2+^ (Figure 3(C1,C2)). In addition, another anti-inflammatory cytokine IL-10 (green fluorescence) expression was upregulated under Mg^2+^ concentrations from 2–32 mM, with the most obvious increase observed in the 8 mM (Figure 3(D1,D2)). Overall, these findings indicate that Mg^2+^ can promote macrophage polarization toward an anti-inflammatory M2 phenotype range from 2 to 32 mM, with optimal immunomodulatory effects observed at 8 mM concentration.

Flow cytometry analysis showed that LPS stimulation markedly increased the proportion of CD86-positive RAW264.7 cells. After Mg^2+^ treatment, the proportion of CD86-positive cells gradually decreased, especially in the 8 mM Mg^2+^ group. However, the inhibitory effect was weakened at 32 mM Mg^2+^. These results suggest that appropriate concentrations of Mg^2+^, especially 8 mM, can attenuate LPS-induced pro-inflammatory macrophage activation (Figure 3E).

### 3.3. The Role of Mg^2+^ on Neuroregulation of RSCs

**Figure 4:**
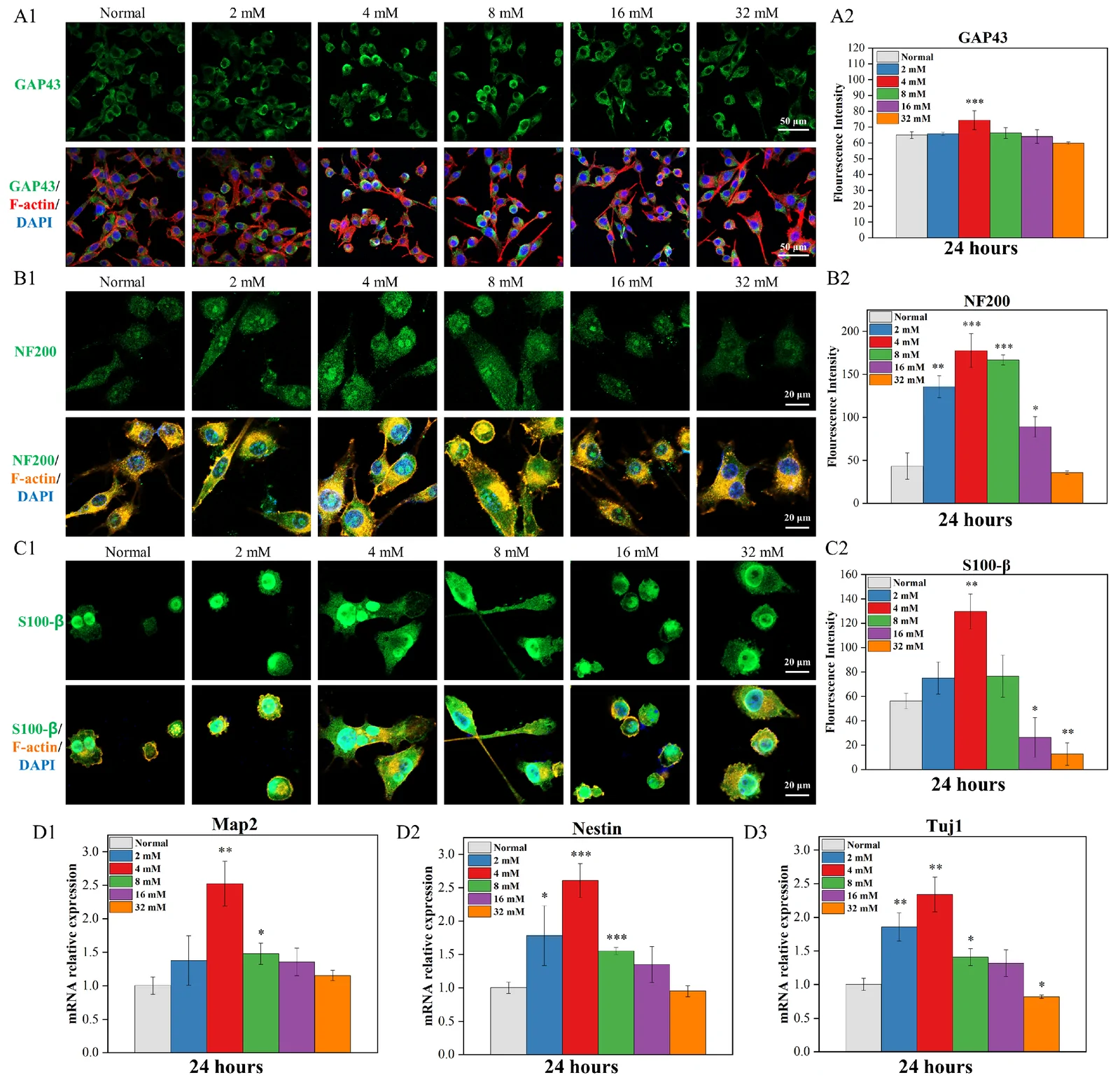
The role of Mg^2+^ on neuroregulation of RSCs. Representative immunofluorescence images of GAP43 (Green, (**A1**)), NF200 (Green, (**B1**)) and S100-β (Green, (**C1**)) in RSCs treated with different concentrations of Mg^2+^ for 24 h. Quantitative fluorescence intensity analysis of GAP43 (**A2**), NF200 (**B2**) and S100-β (**C2**). qPCR analysis of Map2, Nestin, and Tuj1 expression (**D1**–**D3**) in RSCs after Mg^2+^ treatment for 24 h. Data are presented as mean ± SD. *n* = 3. *, significant difference compared to the Normal group, * *p* < 0.05, ** *p* < 0.01, *** *p* < 0.001.

Immunofluorescence staining images and quantitative fluorescence intensity analysis of GAP43, NF200, and S100-β expression of RSCs cultured with Mg^2+^ for 24 h were showed in Figure 4(A1–C2). Among the tested concentrations, 4 mM Mg^2+^ induced improved GAP43 and S100-β expression mostly, while NF200 expression was markedly increased from 2 to 8 mM Mg^2+^ treatment. However, higher Mg^2+^ concentrations, especially ranges from 16 to 32 mM, did not further enhance these nerve repair-related markers. Consistently, qPCR analysis showed that the expression levels of Map2, Nestin, and Tuj1 were upregulated after Mg^2+^ treatment, with obvious expression observed in the 4 mM group (Figure 4(D1–D3)). These results suggest that moderate Mg^2+^ concentrations, particularly 4 mM, may promote the nerve repair-related phenotype of RSCs.

### 3.4. Regulatory Effects of Mg^2+^ on Angiogenesis of HUVECs

**Figure 5:**
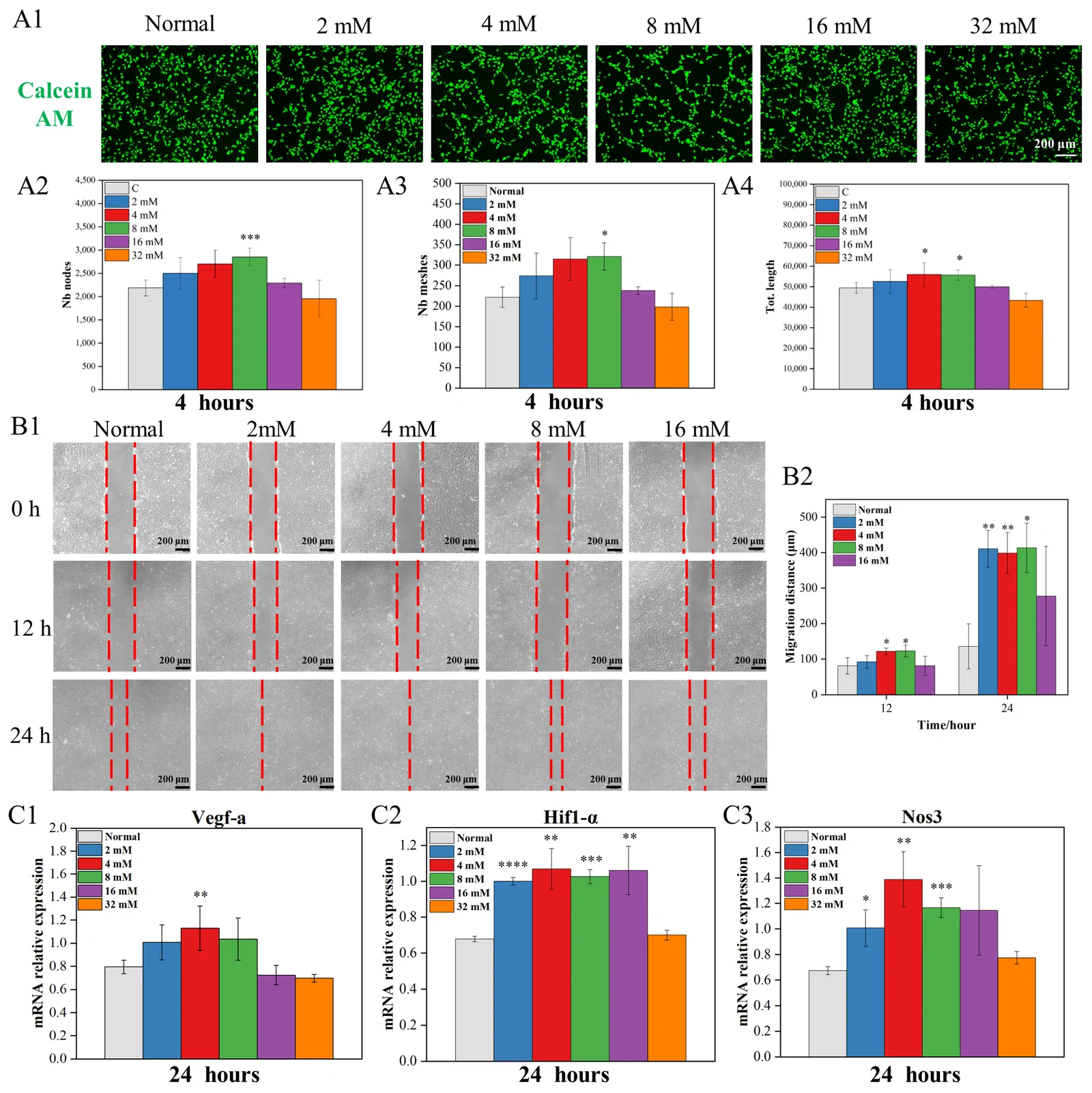
Regulatory effects of Mg^2+^ on angiogenesis of HUVECs. Representative Calcein AM fluorescence images of tube formation (**A1**) and quantitative analysis of tube number, node number, and total tube length (**A2**–**A4**) of HUVECs treated with different concentrations of Mg^2+^. Representative images of wound healing assays (**B1**) and quantitative analysis of migration distance (**B2**) after Mg^2+^ treatment at 0, 12, and 24 h. qPCR analysis of Vegf-a, Hif1-α, and Nos3 expression after 24 h of Mg^2+^ treatment (**C1**–**C3**). Data are presented as mean ± SD. *n* = 3. *, significant difference compared to the Normal group, * *p* < 0.05, ** *p* < 0.01, *** *p* < 0.001, **** *p* < 0.0001.

To evaluate the effect of Mg^2+^ on angiogenesis and cell migration, HUVECs were treated with different concentrations of Mg^2+^. Calcein AM staining images showed that moderate Mg^2+^ concentrations (2–8 mM) enhanced tube formation, with increased tube number, node number, and total tube length. Among the tested concentrations, 8 mM Mg^2+^ showed the best pro-angiogenic effect (Figure 5(A1–A4)). Wound healing assays showed that Mg^2+^ treatment promoted cell migration at 12 and 24 h, especially in the range of 2 to 8 mM groups (Figure 5(B1,B2)). qPCR analysis further showed that Mg^2+^ treatment regulated the expression of angiogenesis-related genes in HUVECs (Figure 5(C1–C3)). Compared with the Normal group, Vegf-a expression was upregulated obviously in the 4 mM group, while Hif1-α expression increased after treatment with 2–16 mM Mg^2+^. In addition, Nos3 expression was also upregulated in the 2–8 mM Mg^2+^ groups. These results suggest that appropriate Mg^2+^ concentrations (8 mM) promote angiogenic activity and migratory capacity in HUVECs.

### 3.5. Effect of Mg^2+^ on Osteogenic Activity of hBMSC

**Figure 6:**
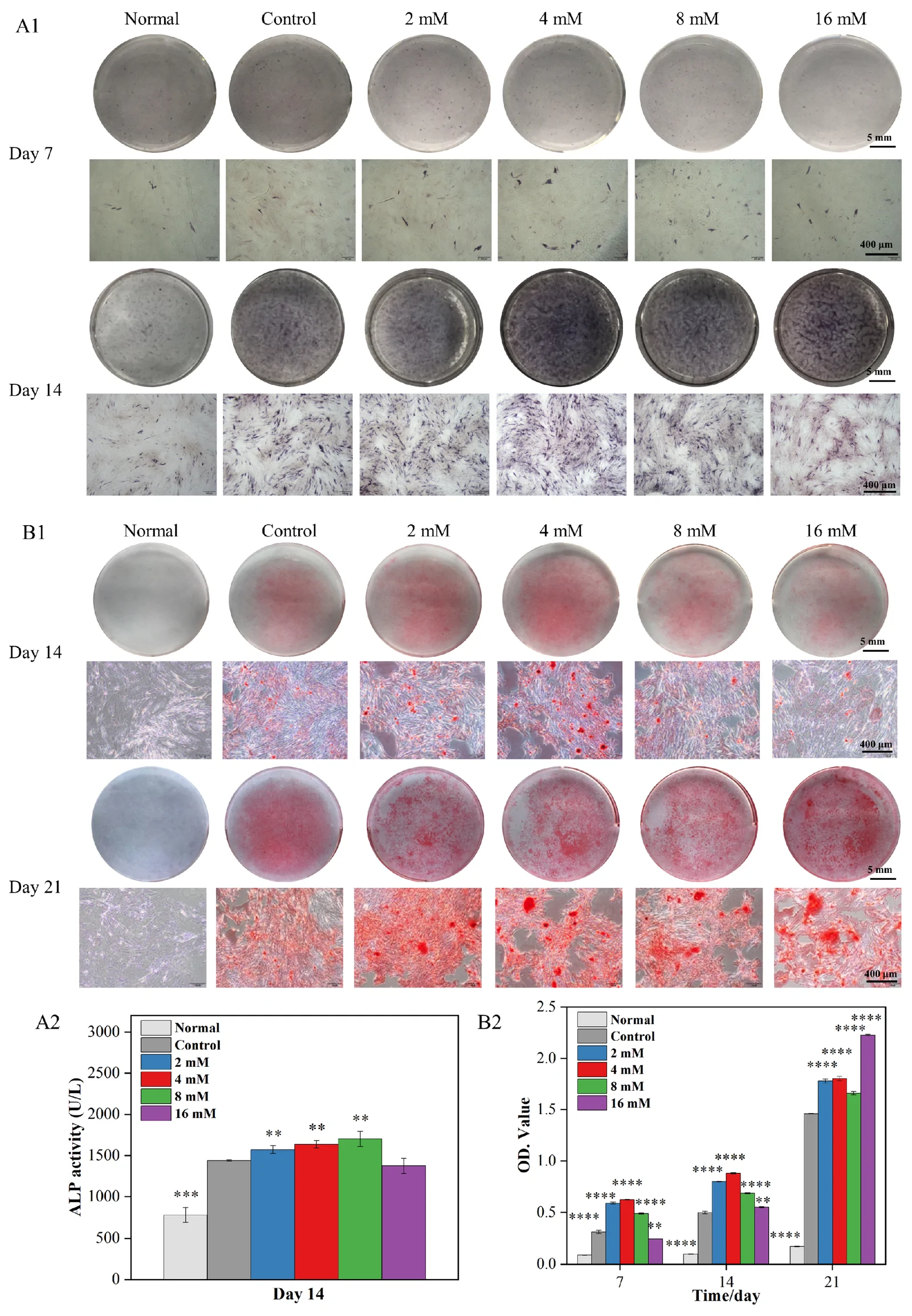
Effect of Mg^2+^ on osteogenic activity of hBMSC. Alkaline phosphatase (ALP) staining images and ALP activity after 7 and 14 days osteogenic induction (**A1**,**A2**) and Alizarin Red S (ARS) staining after 14 and 21 days of osteogenic induction (**B1**). Quantitative analysis of ARS staining was used to assess mineralization (**B2**). Data are presented as mean ± SD. *n* = 3. Significant difference compared to the Control group, ** *p* < 0.01, *** *p* < 0.001, **** *p* < 0.0001.

To evaluate the effects of Mg^2+^ ranging from 0 to 32 mM on the osteogenic differentiation of hBMSC, alkaline phosphatase (ALP) staining, and ALP activity assays, and alizarin Red S (ARS) staining were performed under different Mg^2+^ concentrations. ALP staining results exhibited increased staining intensity at day 7 and 14 of osteogenic induction treated with Mg^2+^ (2–8 mM) (Figure 6(A1)). Furthermore, ALP activity assay demonstrated that hBMSCs treated with Mg^2+^ at 2 to 8 mM have significantly higher ALP activity compared with the Normal group and Control group (Figure 6(A2)). ARS staining results demonstrated that all Mg^2+^-treated groups exhibited more mineralized nodule formation compared with the normal and control group at day 14 and 21 of osteogenic induction. Notably, the 4 mM group exhibited strong calcium deposition as early as day 14 and showed the most abundant and uniformly distributed mineralized nodules at day 21. The 16 mM group did not further enhance mineralization and even showed a slight reduction (Figure 6(B1,B2)).

### 3.6. Mg^2+^ Promote Osteogenic Protein and Gene Expression

**Figure 7:**
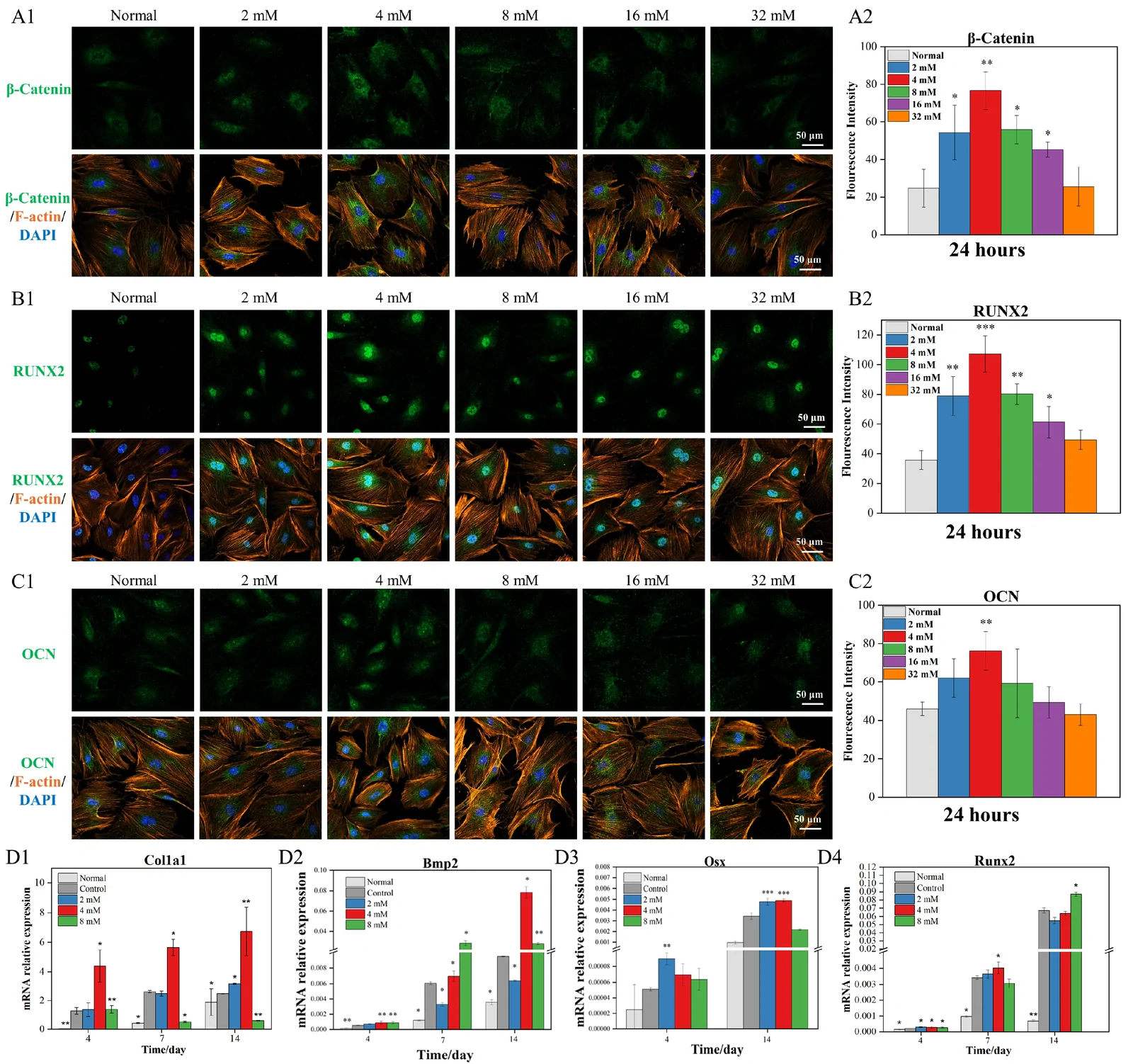
Effects of Mg^2+^ (0–32 mM) on osteogenic differentiation-related markers in hBMSCs. Representative immunofluorescence images and quantitative fluorescence intensity analysis of β-catenin (Green, (**A1**,**A2**)), RUNX2 (Green, (**B1**,**B2**)) and OCN (Green, (**C1**,**C2**)) for 24 h of culture. *n* = 3. *, significant difference compared to the Normal group; qPCR analysis of Col1a1, Bmp2, Osx, and Runx2 expression after treated with Mg^2+^ (0–32 mM) for osteogenic induction at day 4, 7 sand 14 (**D1**–**D4**). Data are presented as mean ± SD. *n* = 3. *, significant difference compared to the Control group, * *p* < 0.05, ** *p* < 0.01, *** *p* < 0.001.

After treatment with different concentrations of Mg^2+^ for 24 h, the expression levels of the osteogenesis-related markers (Runx2 and Ocn) were upregulated. Among the tested concentrations, 4 mM Mg^2+^ markedly increased in both Runx2 and Ocn expression, indicating that this concentration had the strongest stimulatory effect on osteogenesis-related gene expression in hBMSCs (Figure 7(B1–C2)). More interesting, treated with 2–8 mM Mg^2+^ markedly increased β-catenin protein expression in hBMSCs, with the most obvious activation observed in the 4 mM Mg^2+^ group. As β-catenin is a key downstream effector of the canonical Wnt/β-catenin signaling pathway, the increased β-catenin expression suggests that Mg^2+^ may promote the activation of this osteogenesis-related pathway (Figure 7(A1,A2)).

Quantitative PCR analysis revealed distinct concentration-dependent effects of Mg^2+^ on the expression of osteogenic markers (Col1a1, Bmp2, Runx2, Osx) in hBMSCs over 4, 7, and 14 days of culture. Col1a1 expression was significantly upregulated in the 4 mM group on day 4 and day 7, with a further increase on day 14 (Figure 7(D1)). BMP2 expression was significantly elevated in the 4 mM group on day 7 and day 14 (Figure 7(D2)). Osterix (Osx) expression was significantly increased in the 4 mM group on day 4 and day 14 (Figure 7(D3)). Runx2 expression was significantly upregulated in the 4 mM group on day 7 and day 14, with a similar trend in the 8 mM group at day 14 (Figure 7(D4)). These results demonstrate that Mg^2+^ promote osteogenic differentiation in hBMSCs.

## 4. Discussion

Bone repair is a highly coordinated and stage-dependent regenerative process involving inflammatory regulation, neurovascular reconstruction, osteogenic differentiation, and subsequent matrix mineralization. Although hBMSCs, HUVECs, RSCs, and RAW264.7 were used to evaluate osteogenesis, angiogenesis, neuroregulation, and immunomodulation, respectively, these isolated models cannot fully reproduce the complex multicellular interactions that occur during bone repair in vivo. In the present study, Mg^2+^ were shown to exert a broad pro-regenerative effect on multiple cell types involved in bone healing, including macrophages, neural-related cells, endothelial cells, and mesenchymal stem cells. Importantly, these biological effects were not simply dose-dependent in a linear manner, but exhibited a clear concentration- and time-dependent pattern, particularly around 4 mM, producing the most favorable effects.

During the early inflammatory phase of bone repair, an appropriate immune microenvironment is essential for initiating tissue regeneration. Excessive or prolonged M1-type inflammation can impair stem cell recruitment, angiogenesis, and osteogenic differentiation, whereas timely transition toward an anti-inflammatory and pro-reparative M2 phenotype facilitates tissue remodeling and bone regeneration. In this study, LPS stimulated RAW264.7 were used to mimic an inflammatory microenvironment. Mg^2+^ treatment reduced the expression of the M1 macrophage marker iNOS, TNF-α and increased the expression of the M2 marker CD206, IL10, especially at moderate concentrations. These findings indicate that Mg^2+^ can attenuate the pro-inflammatory macrophage phenotype and promote macrophage polarization toward a reparative M2-like state. This immunomodulatory effect may contribute to the resolution of inflammation and create a more favorable microenvironment for subsequent neurovascular repair and osteogenesis.

Following the inflammatory phase, neurovascular reconstruction plays a critical role in successful bone healing. Nerve fibers and blood vessels are closely coupled during bone regeneration, and their coordinated restoration provides trophic factors, oxygen, nutrients, and regulatory signals required for osteogenesis cell recruitment and bone matrix formation. In neural-related RSCs, Mg^2+^ promoted early cell viability and proliferative activity at low to moderate concentrations, while prolonged stimulation or high Mg^2+^ concentrations showed reduced benefits. More importantly, immunofluorescence staining demonstrated that Mg^2+^ enhanced GAP43 expression in RSCs, with the strongest fluorescence intensity observed in the 4 mM group. GAP43 is closely associated with axonal growth, neural remodeling, and nerve regeneration. Therefore, the upregulation of GAP43 suggests that Mg^2+^ may promote a neuroregenerative phenotype in RSCs, thereby supporting neural repair during bone regeneration.

In parallel with neural repair, angiogenesis is indispensable for bone regeneration. Vascular networks not only provide metabolic support but also guide the recruitment of osteogenic cells and establish a regenerative niche for new bone formation. Live/dead staining showed that Mg^2+^ ranging from 0 to 32 mM did not induce obvious cytotoxicity at early time points, indicating good cytocompatibility toward endothelial cells. CCK-8 results further suggested that Mg^2+^ promoted HUVEC proliferative activity at day 1, whereas prolonged stimulation at days 3 and 7 reduced OD values. Consistently, tube formation assays demonstrated that Mg^2+^ significantly enhanced the formation of capillary-like structures, especially at intermediate concentrations of approximately 4 mM. In addition, qPCR analysis showed that Vegf-a, Hif-1α and Nos3 expression were upregulated after Mg^2+^ stimulation, particularly at 4 mM. These results collectively indicate that Mg^2+^ enhances endothelial angiogenic function, thereby supporting vascularized bone repair.

In the osteogenic phase, Mg^2+^ markedly promoted hBMSC osteogenic differentiation and matrix mineralization. ALP staining and ALP activity assays showed enhanced early osteogenic differentiation in Mg^2+^-treated groups, with the strongest effect observed in the 4 mM group. ARS staining further demonstrated increased mineralized nodule formation at later stages, indicating that Mg^2+^ not only promotes early osteogenic commitment but also enhances extracellular matrix mineralization. At the molecular level, Mg^2+^ upregulated several key osteogenesis-related genes, including Col1a1, Bmp2, Runx2, and Osx. Consistent with these findings, immunofluorescence staining further confirmed the pro-osteogenic effect of Mg^2+^ on hBMSCs. The expression levels of RUNX2 and OCN were markedly increased after Mg^2+^ treatment, with the most evident enhancement observed in the 4 mM Mg^2+^ group. In addition, β-catenin immunofluorescence staining showed increased β-catenin expression after Mg^2+^ treatment, particularly at 4 mM, suggesting that the pro-osteogenic effect of Mg^2+^ may be associated with activation of β-catenin-related signaling. However, this result should be interpreted as supportive evidence rather than definitive pathway validation, and further mechanistic studies are required to confirm the involvement of the Wnt/β-catenin pathway.

Taken together, these findings suggest that Mg^2+^ promotes bone repair through a sequential and coordinated regulatory mechanism. In the early stage, Mg^2+^ modulates macrophage polarization by suppressing the inflammatory M1 phenotype and promoting M2-like reparative polarization. In the intermediate reparative phase, Mg^2+^ supports neurovascular reconstruction by enhancing GAP43 expression in RSCs, promoting endothelial angiogenic activity, increasing Vegf-a expression, and improving hBMSC migration. In the late osteogenic phase, Mg^2+^ enhances osteogenic differentiation and mineralized matrix deposition by upregulating osteogenic markers and increasing ALP activity and calcium deposition. Therefore, Mg^2+^ functions as a multifunctional bioactive ion that integrates immunomodulation, neurovascular repair, stem cell recruitment, and osteogenesis during bone regeneration.

Importantly, the present results also highlight the necessity of optimizing Mg^2+^ concentration for bone repair applications. Although low to moderate concentrations of Mg^2+^ exhibited favorable biological effects, excessive Mg^2+^ exposure showed reduced promotive effects or even inhibitory trends in several assays. These findings suggest that controlled Mg^2+^ release may be important for regulating bone-regeneration-related cellular responses. Among the tested concentrations, 8 mM Mg^2+^ exhibited the optimal immunomodulatory effect on macrophages, while 4 mM Mg^2+^ showed favorable effects on several in vitro cellular processes, including osteogenesis differentiation, angiogenesis-related responses, neural repair-related marker expression. However, this concentration should not be interpreted as a definitive optimal dose for in vivo bone repair, since bone regeneration involves complex multicellular interactions, biomaterial degradation, local ion diffusion, vascularization, immune responses, and tissue remodeling. Therefore, the present results provide preliminary in vitro reference information for the future design of Mg^2+^-releasing biomaterials, and further validation using release-controlled biomaterial systems.

## 5. Conclusions

In this study, the biological effects of Mg^2+^ were interpreted from the perspective of time-sequential regulation during bone regeneration. Bone repair is a dynamic process involving overlapping stages, including inflammatory regulation, angiogenesis, neuroregulation, osteogenic differentiation, and matrix mineralization. The results indicate that different stages of bone regeneration may require different Mg^2+^ concentration ranges. During the early inflammatory phase, moderate Mg^2+^ concentrations (4–16 mM), particularly 8 mM, exhibited pronounced immunomodulatory effects on LPS-stimulated macrophages by increasing IL-10 expression, decreasing TNF-α expression, and reducing CD86-positive pro-inflammatory macrophages. These results suggest that an Mg^2+^-enriched microenvironment during the initial stage may contribute to the resolution of excessive inflammation and facilitate the transition toward a reparative immune phenotype. As biomaterial degradation proceeds and local Mg^2+^ concentrations gradually decline, lower to moderate Mg^2+^ levels, especially 2–8 mM Mg^2+^, may be more favorable for middle to late-stage bone regeneration-related processes, including osteogenic differentiation of hBMSC, angiogenic activity of HUVEC, and neuroregulation of RSCs. Collectively, these findings support the concept that the biological activity of Mg^2+^ is concentration- and stage-dependent during bone regeneration. Therefore, instead of pursuing a constant Mg^2+^ concentration, the rational design of Mg-containing biomaterials may benefit from programmable and time-sequential Mg^2+^ release profiles. An initial release maintaining approximately 4–8 mM Mg^2+^ may be advantageous for immunomodulation and inflammatory regulation, followed by a sustained release at approximately 0–4 mM to support angiogenesis, osteogenesis, and neuroregeneration. This stage-specific strategy may provide a useful design principle for developing Mg-based biomaterials with enhanced regenerative efficacy.

## Figures and Tables

**Table 1:** Source information of cells used in this study.

Cell Type	Species	Source	Catalog No.
hBMSCs	Human	Purchased from Procell Company, China	CP-H166
HUVECs	Human	Purchased from Procell Company, China	CP-H082
RAW264.7	Mouse	Purchased from Procell Company, China	CL-0190
RSCs	Rat	Purchased from Procell Company, China	CL-0199

**Table 2:** List of antibodies used in this study.

Antibody	Host Species	Isotypes	Conjugations	Co. Cat
CD206	Rabbit	IgG	unconjugated	HUABIO. ET1702-04
iNOS	Rabbit	IgG	unconjugated	HUABIO. HA721583
GAP43	Rabbit	IgG	unconjugated	HUABIO. ET1610-94
β-Catenin	Rabbit	IgG	unconjugated	HUABIO. ET1601-5
RUNX2	Rabbit	IgG	unconjugated	HUABIO. ET1612-47
OCN	Rabbit	IgG	unconjugated	HUABIO. ER1919-20
IL-10	Rabbit	IgG	unconjugated	HUABIO. HA722032
TNF-α	Rabbit	IgG	unconjugated	HUABIO. ET1703-63
NF200	Rabbit	IgG	unconjugated	HUABIO. HA723977
S100β	Rabbit	IgG	unconjugated	HUABIO. HA723900

**Table 3:** Primer sequences for qPCR.

Target Gene	Primer Type	Primer Sequence (5′–3′)
*Runx2*	Forward primer	GACACCATGTCAGCAAAACTTCT
	Reverse primer	GGGTGGTCGGCGATGATCT
*Osx*	Forward primer	TCTGCGGGACTCAACAACTC
	Reverse primer	TAGCATAGCCTGAGGTGGGT
*Col1* *A1*	Forward primer	GAGGAGAGCCTGGCAACATT
	Reverse primer	AGGACCAGGGAGACCAAACT
*B* *mp* *2*	Forward primer	AGTGGGAAAACAACCCGGAG
	Reverse primer	TGGTCACGGGGAATTTCGAG
*V* *egf-a*	Forward primer	CTGGAGCGTGTACGTTGGT
	Reverse primer	GCAACGCGAGTCTGTGTTTTT
*Hif1α*	Forward primer	TATGAGCCAGAAGAACTTTTAGGC
	Reverse primer	CACCTCTTTTGGCAAGCATCCTG
*Nos3*	Forward primer	GAAGGCGACAATCCTGTATGGC
	Reverse primer	TGTTCGAGGGACACCACGTCAT
*Tuj1*	Forward primer	GCAACTATGTGGGGGACTCG
	Reverse primer	CAGCACCACTCTGACCGAAGA
*Map2*	Forward primer	ACTGGAAGAAGCCTCGAAGATG
	Reverse primer	TACTTGTGTCCGGCTGAGGA
*Nestin*	Forward primer	AGCACTCCCATCCCACCTAT
	Reverse primer	GGGTTGTGGCTAAGGAGGTC
*h-* *GAPDH*	Forward primer	GAGAAGGCTGGGGCTCATTT
	Reverse primer	AGTGATGGCATGGACTGTGG
*Rat-GAPDH*	Forward primer	CAACTCCCTCAAGATTGTCAGCAA
	Reverse primer	GGCATGGACTGTGGTCATGA

## Data Availability

The data supporting the findings of this study are included within the article. Additional raw data are available from the corresponding author upon reasonable request.

## References

[1] Xu Z., Deng J., Gao D., Du Y., Zhang Y., Lai Y. (2025). Stimuli-responsive biomedical polymeric films for tissue regeneration. Microstructures.

[2] Li J., Ke H., Wu D., Shao Y., Wen Z., Zhong S., Lei X., Li J., Xie D., Zeng C. (2025). Bilayer book- like decellularized extracellular matrix scaffold with bioactive coatings for rotator cuff repair. Mater. Today Bio.

[3] Du Y., Liu Y., Zhang Y., Nie Y., Xu Z., Qin L., Zhang W., Lai Y. (2025). Structurally and Functionally Adaptive Biomimetic Periosteum: Materials, Fabrication, and Construction Strategies. Exploration.

[4] Xie Y., Dong C., Liu Z., Yi Y., Zhou Y. (2025). The Influence of Rolling Reduction on the Mechanical, Corrosion, Osteogenic, and Antibacterial Properties of Zn-Mg Alloys. ACS Omega.

[5] Wang S., Gao Y., Dong L., Shu J., Feng F., Ren L., Hu X., Yang L., Lei M., Chen X. (2026). Acid neutralization and metal-phenolic nanomedicine on-demand release microspheres for reversing cartilage degeneration. J. Control. Release.

[6] Xie Q., Du X., Liang J., Shen Y., Ling Y., Huang Z., Ke Z., Li T., Song B., Wu T. (2025). FABP4 inhibition suppresses bone resorption and protects against postmenopausal osteoporosis in ovariectomized mice. Nat. Commun..

[7] Zhang W., Li L., Wang Z., Nie Y., Yang Y., Li C., Zhang Y., Jiang Y., Kou Y., Zhang W. (2025). Injectable and adhesive MgO2-potentiated hydrogel with sequential tumor synergistic therapy and osteogenesis for challenging postsurgical osteosarcoma treatment. Biomaterials.

[8] Azimi R., Shahgholi M., Khandan A. (2024). Fabrication and characterization of reinforced glass ionomer cement by zinc oxide and hydroxyapatite nanoparticles. Heliyon.

[9] Zhang X., Zu H., Zhao D., Yang K., Tian S., Yu X., Lu F., Liu B., Yu X., Wang B. (2017). Ion channel functional protein kinase TRPM7 regulates Mg ions to promote the osteoinduction of human osteoblast via PI3K pathway: In vitro simulation of the bone-repairing effect of Mg-based alloy implant. Acta Biomater..

[10] Zhang J., Tang L., Qi H., Zhao Q., Liu Y., Zhang Y. (2019). Dual Function of Magnesium in Bone Biomineralization. Adv. Healthc. Mater..

[11] Yoshizawa S., Brown A., Barchowsky A., Sfeir C. (2014). Magnesium ion stimulation of bone marrow stromal cells enhances osteogenic activity, simulating the effect of magnesium alloy degradation. Acta Biomater..

[12] Yoshizawa S., Brown A., Barchowsky A., Sfeir C. (2014). Role of magnesium ions on osteogenic response in bone marrow stromal cells. Connect. Tissue Res..

[13] Wang J., Ma X.-Y., Feng Y.-F., Ma Z.-S., Ma T.-C., Zhang Y., Li X., Wang L., Lei W. (2017). Magnesium Ions Promote the Biological Behaviour of Rat Calvarial Osteoblasts by Activating the PI3K/Akt Signalling Pathway. Biol. Trace Elem. Res..

[14] Qi T., Weng J., Yu F., Zhang W., Li G., Qin H., Tan Z., Zeng H. (2021). Insights into the Role of Magnesium Ions in Affecting Osteogenic Differentiation of Mesenchymal Stem Cells. Biol. Trace Elem. Res..

[15] Li C., Zhang W., Nie Y., Jiang D., Jia J., Zhang W., Li L., Yao Z., Qin L., Lai Y. (2023). Integrated and Bifunctional Bilayer 3D Printing Scaffold for Osteochondral Defect Repair. Adv. Funct. Mater..

[16] Li C., Zhang W., Wang R., Du X.-F., Jiang D., Liu B., Nie Y., Liao J., Chen Y., Liang X. (2022). Nanocomposite multifunctional hydrogel for suppressing osteosarcoma recurrence and enhancing bone regeneration. Chem. Eng. J..

[17] Liu W., Guo S., Tang Z., Wei X., Gao P., Wang N., Li X., Guo Z. (2020). Magnesium promotes bone formation and angiogenesis by enhancing MC3T3-E1 secretion of PDGF-BB. Biochem. Biophys. Res. Commun..

[18] Li Y., Wang J., Yue J., Wang Y., Yang C., Cui Q. (2018). High magnesium prevents matrix vesicle-mediated mineralization in human bone marrow-derived mesenchymal stem cells via mitochondrial pathway and autophagy. Cell Biol. Int..

[19] Li R.W., Kirkland N.T., Truong J., Wang J., Smith P.N., Birbilis N., Nisbet D.R. (2014). The influence of biodegradable magnesium alloys on the osteogenic differentiation of human mesenchymal stem cells. J. Biomed. Mater. Res. Part A.

[20] Kim K.-J., Choi S., Sang Cho Y., Yang S.-J., Cho Y.-S., Kim K.K. (2017). Magnesium ions enhance infiltration of osteoblasts in scaffolds via increasing cell motility. J. Mater. Sci. Mater. Med..

[21] Hung C.-C., Chaya A., Liu K., Verdelis K., Sfeir C. (2019). The role of magnesium ions in bone regeneration involves the canonical Wnt signaling pathway. Acta Biomater..

[22] He L.Y., Zhang X.M., Liu B., Tian Y., Ma W.H. (2016). Effect of magnesium ion on human osteoblast activity. Braz. J. Med. Biol. Res..

[23] Wang M., Yu Y., Dai K., Ma Z., Liu Y., Wang J., Liu C. (2016). Improved osteogenesis and angiogenesis of magnesium-doped calcium phosphate cement via macrophage immunomodulation. Biomater. Sci..

[24] Wang L., Wang X., Wu J., Chen J., He Z., Wang J., Zhang X. (2025). Magnesium Ions Induce Endothelial Cell Differentiation into Tip Cell and Enhance Vascularized Bone Regeneration. Adv. Healthc. Mater..

[25] Wang L., Shen M., Tang Z., Tan J., Li K., Ma H. (2025). 3D printed magnesium silicate/β-tricalcium phosphate scaffolds promote coupled osteogenesis and angiogenesis. Front. Bioeng. Biotechnol..

[26] Liu L., Wang F., Song W., Zhang D., Lin W., Yin Q., Wang Q., Li H., Yuan Q., Zhang S. (2024). Magnesium promotes vascularization and osseointegration in diabetic states. Int. J. Oral Sci..

[27] Liu C., Yang G., Zhou M., Zhang X., Wu X., Wu P., Gu X., Jiang X. (2021). Magnesium Ammonium Phosphate Composite Cell-Laden Hydrogel Promotes Osteogenesis and Angiogenesis In Vitro. ACS Omega.

[28] Liu C., Fu X., Pan H., Wan P., Wang L., Tan L., Wang K., Zhao Y., Yang K., Chu P.K. (2016). Biodegradable Mg-Cu alloys with enhanced osteogenesis, angiogenesis, and long-lasting antibacterial effects. Sci. Rep..

[29] Lai Y., Li Y., Cao H., Long J., Wang X., Li L., Li C., Jia Q., Teng B., Tang T. (2019). Osteogenic magnesium incorporated into PLGA/TCP porous scaffold by 3D printing for repairing challenging bone defect. Biomaterials.

[30] Hu J., Huang G., Li L., Zhan X., Zhang J., Shao J., Hong S., Pan S.-T. (2024). A Mg^2+^-light double crosslinked injectable alginate hydrogel promotes vascularized osteogenesis by establishing a Mg^2+^-enriched microenvironment. Mater. Today Commun..

[31] Ho C.-C., Hsu T.-T., Chiu Y.-C., Lin Y.-H., Xie P.-C., Wang C.-Y. (2025). 3D-printed magnesium/strontium-co-doped calcium silicate scaffolds promote angiogenesis and bone regeneration through synergistic bioactive ion stimulation. J. Biol. Eng..

[32] Xu L., Willumeit-Römer R., Luthringer-Feyerabend B.J.C. (2019). Effect of magnesium-degradation products and hypoxia on the angiogenesis of human umbilical vein endothelial cells. Acta Biomater..

[33] Helmholz H., Resuli R., Tacke M., Nourisa J., Tomforde S., Aydin R., Willumeit-Römer R., Zeller-Plumhoff B. (2025). Regression models for the prediction of the influence of magnesium ions on primary endothelial cell (HUVEC) proliferation and migration. Comput. Struct. Biotechnol. J..

[34] Sreenivasamurthy S.A., Akhter F.F., Akhter A., Su Y., Zhu D. (2022). Cellular mechanisms of biodegradable zinc and magnesium materials on promoting angiogenesis. Biomater. Adv..

[35] Li C., Zhang W., Nie Y., Du X., Huang C., Li L., Long J., Wang X., Tong W., Qin L. (2024). Time-Sequential and Multi-Functional 3D Printed MgO2/PLGA Scaffold Developed as a Novel Biodegradable and Bioactive Bone Substitute for Challenging Postsurgical Osteosarcoma Treatment. Adv. Mater..

[36] Zhou H., He Z., Cao Y., Chu L., Liang B., Yu K.-X., Deng Z. (2024). An injectable magnesium-loaded hydrogel releases hydrogen to promote osteoporotic bone repair via ROS scavenging and immunomodulation. Theranostics.

[37] Zhang X., Chen Q., Mao X. (2019). Magnesium Enhances Osteogenesis of BMSCs by Tuning Osteoimmunomodulation. BioMed Res. Int..

[38] Wang Q., Xu L., Willumeit-Römer R., Luthringer-Feyerabend B.J.C. (2021). Macrophage-derived oncostatin M/bone morphogenetic protein 6 in response to Mg-based materials influences pro-osteogenic activity of human umbilical cord perivascular cells. Acta Biomater..

[39] Wang Q., Xu L., Helmholz H., Willumeit-Römer R., Luthringer-Feyerabend B.J.C. (2020). Effects of degradable magnesium on paracrine signaling between human umbilical cord perivascular cells and peripheral blood mononuclear cells. Biomater. Sci..

[40] Timofticiuc I.-A., Călinescu O., Iftime A., Dragosloveanu S., Caruntu A., Scheau A.-E., Badarau I.A., Didilescu A.C., Caruntu C., Scheau C. (2024). Biomaterials Adapted to Vat Photopolymerization in 3D Printing: Characteristics and Medical Applications. J. Funct. Biomater..

[41] Tan S., Wang Y., Du Y., Xiao Y., Zhang S. (2021). Injectable bone cement with magnesium-containing microspheres enhances osteogenesis via anti-inflammatory immunoregulation. Bioact. Mater..

[42] Qiao W., Wong K.H.M., Shen J., Wang W., Wu J., Li J., Lin Z., Chen Z., Matinlinna J.P., Zheng Y. (2021). TRPM7 kinase-mediated immunomodulation in macrophage plays a central role in magnesium ion-induced bone regeneration. Nat. Commun..

[43] Hu T., Xu H., Wang C., Qin H., An Z. (2018). Magnesium enhances the chondrogenic differentiation of mesenchymal stem cells by inhibiting activated macrophage-induced inflammation. Sci. Rep..

[44] Hang R., Tian X., Qu G., Zhao Y., Yao R., Zhang Y., Wei W., Yao X., Chu P.K. (2022). Exosomes derived from magnesium ion-stimulated macrophages inhibit angiogenesis. Biomed. Mater..

[45] Kirkland A.E., Sarlo G.L., Holton K.F. (2018). The Role of Magnesium in Neurological Disorders. Nutrients.

[46] Bhat K., Schlotterose L., Hanke L., Helmholz H., Quandt E., Hattermann K., Willumeit-Römer R. (2024). Magnesium-lithium thin films for neurological applications-An in vitro investigation of glial cytocompatibility and neuroinflammatory response. Acta Biomater..

[47] Yao Z., Yuan W., Xu J., Tong W., Mi J., Ho P.-C., Chow D.H.K., Li Y., Yao H., Li X. (2022). Magnesium-Encapsulated Injectable Hydrogel and 3D-Engineered Polycaprolactone Conduit Facilitate Peripheral Nerve Regeneration. Adv. Sci..

[48] Yao Z., Chen Z., He X., Wei Y., Qian J., Zong Q., He S., Song L., Ma L., Lin S. (2025). Bioactive MgO/MgCO3/Polycaprolactone Multi-gradient Fibers Facilitate Peripheral Nerve Regeneration by Regulating Schwann Cell Function and Activating Wingless/Integrase-1 Signaling. Adv. Fiber Mater..

[49] Vennemeyer J.J., Hopkins T., Hershcovitch M., Little K.D., Hagen M.C., Minteer D., Hom D.B., Marra K., Pixley S.K. (2014). Initial observations on using magnesium metal in peripheral nerve repair. J. Biomater. Appl..

[50] Tatu R., White L.G., Yun Y., Hopkins T., An X., Ashraf A., Little K.J., Hershcovitch M., Hom D.B., Pixley S. (2023). Effects of Altering Magnesium Metal Surfaces on Degradation In Vitro and In Vivo during Peripheral Nerve Regeneration. Materials.

[51] Sun Y., Zhang H., Zhang Y., Liu Z., He D., Xu W., Li S., Zhang C., Zhang Z. (2023). Li–Mg–Si bioceramics provide a dynamic immuno-modulatory and repair-supportive microenvironment for peripheral nerve regeneration. Bioact. Mater..

[52] Sun L., Wang M., Chen S., Sun B., Guo Y., He C., Mo X., Zhu B., You Z. (2018). Molecularly engineered metal-based bioactive soft materials—Neuroactive magnesium ion/polymer hybrids. Acta Biomater..

[53] Ramburrun P., Kumar P., Choonara Y.E., du Toit L.C., Pillay V. (2019). Design and characterisation of PHBV-magnesium oleate directional nanofibers for neurosupport. Biomed. Mater..

[54] Pan H.-C., Sheu M.-L., Su H.-L., Chen Y.-J., Chen C.-J., Yang D.-Y., Chiu W.-T., Cheng F.-C. (2011). Magnesium supplement promotes sciatic nerve regeneration and down-regulates inflammatory response. Magnes. Res..

[55] Li B.-H., Yang K., Wang X. (2016). Biodegradable magnesium wire promotes regeneration of compressed sciatic nerves. Neural Regen. Res..

[56] Kim H., Kwon J., Kim H., Lee S., Kim S., Lee J.-Y., Rahaman K.A., Kim T., Lee H., Ok M.-R. (2025). Controlled Magnesium Ion Delivery via Mg-Sputtered Nerve Conduit for Enhancing Peripheral Nerve Regeneration. Adv. Healthc. Mater..

[57] Hopkins T.M., Little K.J., Vennemeyer J.J., Triozzi J.L., Turgeon M.K., Heilman A.M., Minteer D., Marra K., Hom D.B., Pixley S.K. (2017). Short and long gap peripheral nerve repair with magnesium metal filaments. J. Biomed. Mater. Res. Part A.

[58] Li L., Cheng L., Du Y., Zhang Y., Wang Z., Nie Y., Long J., Li C., Zhang Y., Lai Y. (2025). Exosomes Derived from Mg-Preconditioned Bone Mesenchymal Stem Cells Promote Angiogenesis and Osteogenesis for Osteonecrosis Treatment. Materials.

[59] Meng X., Qin L., Wang X. (2025). Biased agonism of G protein-coupled receptors as a novel strategy for osteoarthritis therapy. Bone Res..

[60] Yang Y., Yao Z., Sun Y., Nie Y., Zhang Y., Li Z., Luo Z., Zhang W., Wang X., Du Y. (2025). 3D-printed manganese dioxide incorporated scaffold promotes osteogenic-angiogenic coupling for refractory bone defect by remodeling osteo-regenerative microenvironment. Bioact. Mater..

